# Psychological distress and nutritional status in head and neck cancer patients: a pilot study

**DOI:** 10.1007/s00405-020-05798-y

**Published:** 2020-02-05

**Authors:** Maja Gosak, Kaja Gradišar, Nada Rotovnik Kozjek, Primož Strojan

**Affiliations:** 1 Department of Radiation Oncology, Institute of Oncology, Zaloška 2, 1000 Ljubljana, Slovenia; 2 Clinical Nutrition Unit, Institute of Oncology, Ljubljana, Slovenia; 3 Faculty of Medicine, University of Ljubljana, Ljubljana, Slovenia

**Keywords:** Head and neck cancer, Radiotherapy, Chemotherapy, Nutrition, Psychological status

## Abstract

**Purpose:**

To determine whether the psychological state of patients with head and neck cancer (HCN) is associated with their nutritional status.

**Methods:**

In 40 patients with locally advanced HNC treated with definitive or adjuvant (chemo)radiotherapy, psychological and nutritional status were assessed before treatment, at its completion and 3 months’ post-therapy. Psychosocial distress was measured using the Hospital Anxiety and Depression Scale questionnaire (HADS-A, HADS-D), whereas the nutritional status was evaluated using standard methods (Nutritional Risk Screening Tool 2002, anthropometric data, dynamometry and laboratory tests) and with a bioelectrical impedance analysis parameter phase angle (PA).

**Results:**

Before treatment, more patients were screened positive for anxiety than at treatment completion (*p* = 0.037) or 3 months’ post-therapy (*p* = 0.083). Depression prevalence was non-significantly higher at the end and after therapy. Compared to the baseline, more cachectic patients and a reduction of PA values were found at successive assessments. Anxiety was more often recorded among malnourished/cachectic patients (assessment 1, *p* = 0.017; assessment 2, *p* = 0.020) who were also found more frequently depressed (assessment 2, *p* = 0.045; assessment 3, *p* = 0.023). Significantly higher PA values were measured in patients without distress determined at 3 months’ post-therapy by the HADS-A (*p* = 0.027).

**Conclusion:**

The association between the psychological and nutritional status found in this pilot study and the options for intervention warrants further clarification in a larger prospective trial.

## Introduction

Head and neck cancer (HNC) is the eighth most common cancer worldwide [1]. Despite the increasing etiological importance of human papillomavirus (HPV) infection observed in recent years, the long-lasting history of smoking and alcohol abuse is still the prevailing cause of this cancer, and reflects the lower socioeconomic status of the patients [2]. Consequently, the time from the symptoms’ appearance and the first medical consultation is often delayed in these patients [3]. At presentation, two-thirds of them have a locally advanced disease with associated anatomical abnormalities and functional disorders in the affected area [2].

Facing the diagnosis of a malignant disease adversely influences the psychological profile of patients, which is further aggravated with troublesome symptoms of the disease and long-lasting and aggressive oncological treatment with associated toxic effects [4]. Thus, patients with HNC often face difficulties with social integration, which can lead to isolation and symptoms of depression and anxiety [4, 5]. The risk of developing a mood disorder correlates with the patients’ sex, age and size of tissue destruction, caused by either a tumor or treatment [5–7]. HNC patients were found to suffer from these symptoms more frequently than other cancer patients [8]. Moreover, at 1 year after treatment completion, studies showed the anxiety disorder still in every fifth patient (20%) and the prevalence of depression of 17%, whereas the suicide rate among these patients was more than 3 times higher than in the general population [9, 10].

Nutritional disorders such as malnutrition, micronutrients deficiencies and cachexia are among the main consequences of inflammation and lower food intake that resulted from anatomic and functional changes in the upper aerodigestive tract caused by a tumor or treatment. HNC patients are at a high risk for malnutrition that affects about 50% of them at the time of disease presentation and closely correlates with the morbidity and mortality rates [11–13]. Also, malnutrition increases the risk of acute and late treatment-related complications which may adversely impact treatment efficacy and the chances of a cure [11, 14].

In the literature, there is only limited information on the relationship between the psychological and nutritional status of patients with HNC. Moreover, as pertinent studies were performed in different cultural and social environments, their practical value for other populations may be limited. When evaluating the results of these studies, it is essential to take into consideration the characteristics of the social and cultural environment from which the patients derive, and the associated system of values. Therefore, the aim of our pilot study was to determine whether the psychological state of Slovenian patients with HCN is associated with their nutritional status.

## Patients and methods

### Patient eligibility

Eligible patients were ≥ 18-year-old men with histologically confirmed UICC TNM stage III–IVB (7th ed.) squamous cell carcinoma of the oral cavity, oropharynx, hypopharynx, or larynx, treated with definitive or postoperative (chemo)radiotherapy (RT) with a curative intent and ≥ 50 Gy to the mucosa (inclusion of ≥ 75% of the oral and pharyngo-laryngeal lining) and tissues of both sides of the neck. In patients treated with primary surgery, the surgical procedure comprised the resection of the primary tumor together with a surrounding margin of normal tissue and removal of the related regional lymph nodes on the neck. Patients with simultaneously diagnosed or previously treated malignancy (with the exception of a basal cell carcinoma of the skin), a pacemaker or defibrillator, severe chronic diseases (of the heart, lungs, liver, kidneys, gastrointestinal tract), active uncontrolled infection, neuromuscular disorders, hemiplegia and arthritis affecting the hands, were deemed ineligible due to the potential influence of these conditions to the results of the planned measurements and tests. Patients considered not being able to understand and complete the questionnaires were also excluded.

Linac-based intensity-modulated RT and concurrent weekly cisplatin (40 mg/m^2^ IV, in patients at high-risk for in-field recurrence) were employed as indicated by the Multidisciplinary Head and Neck Tumor Board. Before treatment and weekly during the course of RT, a nutritional status was evaluated and undernourished patients were offered individual dietary counseling. Detailed information on the characteristics of patients (gender, age, co-morbidities), disease (site of the index cancer, histology, TNM stage), and treatment (definitive/postoperative, daily/total RT dose, chemotherapy) was prospectively collected.

### Study design and investigations

The study protocol was approved by the Republic of Slovenia National Medical Ethics Committee (No. 46/02/15) and all patients signed a written informed consent after receiving detailed information about the study.

The study was designed as being prospective, with the planned inclusion of 40 patients who would complete all the planned investigations. In each patient, the psychological and nutritional status were assessed at three time points: 2–3 weeks before commencement of (chemo)RT, at the time of treatment completion, and 3 months’ post-therapy.

The psychosocial distress was measured using the Hospital Anxiety and Depression Scale (HADS) questionnaire, which consists of two 7-item scales, HADS-A and HADS-D, asking patients about their immediate feelings. Each item was scored from 0 to 3 and subscale scores > 7 are indicative of anxiety and depression [15]. The questionnaire was completed by the patients themselves, with two of the investigators (MG, KG) present to answer any queries.

The nutritional status was evaluated using standard methods (Nutritional Risk Screening Tool 2002 [NRS-2002], anthropometric data, dynamometry and laboratory tests) and with bioelectrical impedance analysis (BIA). Patients who collected ≥ 3 points on the 7-point scale by the NRS-2002 with an age-adjustment for patients aged > 70 years were considered malnourished [16]. Cachectic patients were identified using the international consensus criteria, with a simple starvation excluded by the presence of three out of five criteria: decreased muscle strength, low fat-free mass index (FFMI, < 14.6 kg/m^2^), anorexia, fatigue, and abnormal biochemistry indicating inflammation, anemia or hypoalbuminemia [17, 18]. For this purpose, a body mass index (BMI) was calculated (weight/height^2^ [kg/m^2^]) and the hand-grip strength was measured with Jamar^®^ Hand Dynamometer (Warrenville, IL, USA) as described elsewhere [19]. A fifth percentile normative grip strength values stratified by age and gender were used to determine decreased muscle strength [20]. Anemia, hypoalbuminemia or inflammation were diagnosed at concentrations of hemoglobin < 133 g/l, serum albumin < 35 g/l and C-reactive protein (CRP) > 5 mg/l. Based on the measurements and tests results, patients were categorized as well-nourished, malnourished or cachectic.

The BIA was performed according to the standards of the National Health Institute using a Bodystat^®^ Quadscan 4000 (Douglas, GB) as previously described, at an alternating electric current of 0.8 mA and four frequencies (5, 50, 100 and 200 kHz) [19]. The phase angle (PA) and FFMI were calculated at 50 kHz and according to the validated equations [21].

### Statistical methods

The Shapiro–Wilk test was used for the normality testing of numerical data, which were presented as the mean ± standard deviation or as the median (range). Numerical variables were compared using the ANOVA or Kruskal–Wallis test. Categorical variables were presented as frequencies; the frequency distribution among different groups of patients was tested using the Fisher exact test or Chi square test for a trend. For a pair comparison of numerical variables, a paired *t* test or Wilcoxon signed-rank test was employed. The relationship between the numerical variables was tested using a Pearson correlation or Spearman’s rank correlation. All the analyses were performed using the SPSS version 22.0 (Chicago, IL, USA) and two-sided statistical tests, with a *p* value of ≤ 0.05 considered as being statistically significant.

## Results

Between February 2017 and January 2018, 49 patients with locally advanced SCCHN entered the study. Four patients withdrew their consent before completing the study; two patients died during treatment and in three patients, a non-compliance with the inclusion criteria was discovered before starting their therapies. Thus, 40 male patients completed the whole set of study-related investigations at all three time points and their clinical characteristics are presented in Table 1. All patients completed their treatment as planned. The median time interval between assessment 1 and the commencement of RT was 15.15 ± 3.61 days, and between RT completion and assessments 2 and 3, it was 0.75 ± 6.26.74 days and 90.53 ± 8.94 days, respectively.

**Table 1:** Characteristics of patients, tumors and treatment

Characteristic	No. of patients
Age	62.5 (7.61)^a^
Smoking status	
Active smokers^b^	22 (55%)
Former smokers^c^	13 (32.5%)
Non-smokers	5 (12.5%)
Primary tumor site	
Oral cavity	7 (17.5%)
Oropharynx	21 (52.5%)
Hypopharynx	7 (17.5%)
Larynx	5 (12.5%)
TNM stage	
III	7 (17.5%)
IVA	27 (67.5%)
IVB	6 (15%)
HPV related primary tumors^d^	7 (33.3%)
Tracheostomy before RT	6 (15%)
Surgery before RT	17 (42.5%)
RT dose	66.6 Gy (60–70)^e^
Duration of RT	46.9 days (40–55)^e^
Concurrent chemotherapy	
Yes	21 (52.5%)
No. of cycles	6 (3–7)^d^

^a^In years, mean (standard deviation)

^b^20 cigarettes/day for ≥ 10 years

^c^Quite smoking ≥ 12 months ago

^d^Oropharyngeal primary tumors

^e^Median (range)

The mean HADS-A and HADS-D scores collected in all three measurements were 4.23 ± 3.06 (range 0–15) and 4.06 ± 3.17 (range 0–16), respectively. The distribution of patients according to the results of the HADS sub-questionnaires is shown in Table 2. High rates of anxiety were found before treatment, whereas the depression prevalence was higher at the end and after therapy. The statistically significant differences were only recorded in the case of HADS-A: before treatment, statistically significant more patients were screened positive for psychological distress than at treatment completion (*p* = 0.037) and the same trend was seen at assessment 3 (*p* = 0.083).

**Table 2:** Results of the HADS questionnaire

HADS	Assessment 1	Assessment 2	Assessment 3	*p* value^c^
HADS-A^a^	5.07 (3.14)	3.67 (2.30)	3.95 (3.49)	0.009/0.501/0.004
No distress^b^	29 (72.5%)	37 (92.5%)	36 (90%)	0.037/1.000/0.083
Distress^b^	11 (27.5%)	3 (7.5%)	4 (10%)	
HADS-D^a^	3.65 (2.81)	4.56 (2.94)	3.97 (3.69)	0.136/0.286/0.578
No disress^b^	37 (92.5%)	34 (85%)	34 (85%)	0.381/1.000/0.481
Distress^b^	3 (7.5%)	6 (15%)	6 (15%)	

^a^Scores; mean (standard deviation)

^b^Number of patients

^c^Assessment 1 vs. 2/assessment 2 vs. 3/assessment 1 vs. 3

Due to the loss of more than 5% of body weight, 5 (12.5%) patients had a feeding-tube placement before the commencement of therapy and in an additional two patients it was inserted during treatment; at the last assessment, 4 (10%) patients were still feeding-tube dependent. Before, at the end and 3 months after therapy, the nutritional support in the form of commercially available high caloric oral or enteral (via feeding tube) liquid nutritional supplements was used in 6 (15%), 26 (62.5%), and 22 (55%) of the patients, respectively.

The results of NRS-2002, anthropometric and laboratory tests, and PA measurements are shown in Table 3. Before treatment, there were more well-nourished and less cachectic patients than at assessment 2 (*p* = 0.014) or assessment 3 (*p* = 0.140). The PA values were significantly higher at the initial measurement compared to both subsequent measurements (*p* = 0.019 and *p* = 0.005). The pre-treatment mean value of PA (5.18°) was employed as a cut-off to sort the patients into two groups (< 5.18° vs. ≥ 5.18°) but it was found to be unrelated to the nutritional status determined by the standard tests and measurements (*p* > 0.05) (Table 3).

**Table 3:** Result of nutritional testing

Test/status	Assessment 1	Assessment 2	Assessment 3	*p* value^d^
Test				
NRS 2002 (points)^a^	2 (0–4)	2 (0–4)	2 (0–4)	0.107/0.316/0.779
BMI (kg/m^2^)^b^	25.82 (4.96)	24.61 (4.35)	23.84 (4.03)	< 0.001/0.016/< 0.001
FFMI (kg/m^2^)^b^	19.17 (3.32)	18.28 (2.92)	18.10 (2.47)	< 0.000/0.464/0.004
Muscle strength (kg)^b^	34.75 (11.17)	33.86 (11.73)	32.76 (10.22)	0.345/0.269/0.018
Hemoglobin (g/l)^b^	135.25 (14.46)	127.42 (16.38)	130.47 (15.73)	0.003**/**0.160/0.064
CRP (mg/l)^a^	5 (1–75)	16.50 (1–170)	4.50 (0–126)	< 0.001/< 0.001/0.786
Albumin (g/l)^b^	44.15 (4.34)	41.73 (4.53)	44.42 (4.08)	< 0.001/< 0.001/0.961
PA (°)^b^	5.18 (0.88)	4.95 (0.75)	4.86 (0.90)	0.019/0.368/0.005
Nutritional status^c^				
Well-nourished	29 (72.5%)	22 (55%)	26 (65%)	0.014/0.320/0.140
Malnourished	8 (20%)	4 (10%)	4 (10%)	
Cachectic	3 (7.5%)	14 (35%)	10 (25%)	
PA < 5.2°	19 (47.5%)	24 (60%)	25 (62.5%)	0.370/1.000/0.261
PA ≥ 5.2°	21 (52.5%)	16 (40%)	15 (37.5%)	

*BMI* Body mass index, *FFMI* fat-free mass index, *CRP* C-reactive protein, *PA* phase angle

^a^Median (range)

^b^Mean (standard deviation)

^c^Number of patients

^d^Assessment 1 vs. 2/assessment 2 vs. 3/assessment 1 vs. 3

Different clinical factors (age, smoking status, primary tumor site, disease stage, surgery before RT, concurrent chemotherapy, HPV status, and a tracheostomy before RT) were tested to determine their influence on either the nutritional or psychological status of the patients. At treatment completion, all six patients with depressive symptoms had up-front RT (RT vs. surgery: 26.1 vs. 0%, *p* = 0.030) and received concurrent chemotherapy (yes vs. no: 28.6 vs. 0%, 0.021). In respect to the nutritional status, significantly more patients with baseline PA ≥ 5.2° (i.e. at assessment 1) were treated with up-front RT (RT vs. surgery: 69.6 vs. 29.4%, *p* = 0.24) and concurrent chemotherapy (yes vs. no: 71.4 vs. 31.6%, *p* = 0.025).

The correlation between psychological and conventionally determined nutritional status is presented in Table 4. At assessments 1 and 2, the majority of patients without psychological distress revealed by HADS-A were well-nourished, whereas distress was more often recorded among malnourished/cachectic patients (*p* = 0.017 and *p* = 0.020). According to the HADS-D, significantly more malnourished/cachectic patients than well-nourished ones were found distressed at assessments 2 and 3 (*p* = 0.045 and *p* = 0.023). When the results of the HADS-A and HADS-D were compared with the PA values, only a weak negative correlation was recorded at all three assessment points (*p* > 0.05). However, patients with no psychological distress determined at assessment 3 using the HADS-A had statistically significant higher median PA value (4.9, range 2.3–6.5) compared to those with distress (4.1, range 3.6–4.5; *p* = 0.027).

**Table 4:** Relationship between the psychological and nutritional status

Nutritional status	No distress^a^	Distress^a^	*p* value
HADS-A			
Assessment 1			
Well nourished	24 (82.8%)	5 (45.4%)	0.017
Malnourished	4 (13.8%)	4 (36.4%)	
Cachectic	1 (3.4%)	2 (18.2%)	
Assessment 2			
Well nourished	22 (59.5%)	0	0.020
Malnourished	4 (10.8%)	0	
Cachectic	11 (29.7%)	3 (100%)	
Assessment 3			
Well nourished	24 (66.7%)	2 (50%)	0.327
Malnourished	4 (11.1%)	0	
Cachectic	8 (22.2%)	2 (50%)	
HADS-D			
Assessment 1			
Well nourished	27 (73%)	2 (66.7%)	0.353
Malnourished	8 (21.6%)	0	
Cachectic	2 (5.4%)	1 (33.3%)	
Assessment 2			
Well nourished	21 (61.8%)	1 (16.7%)	0.045
Malnourished	3 (8.8%)	1 (16.7%)	
Cachectic	10 (29.4%)	4 (66.6%)	
Assessment 3			
Well nourished	24 (70.6%)	2 (33.3%)	0.023
Malnourished	4 (11.8%)	0	
Cachectic	6 (17.6%)	4 (66.7%)	

^a^Number of patients

## Discussion

In the present study, in the cohort of Slovenian HNC patients treated with (chemo)RT we found that their psychological and nutritional status were interconnected. Because of possible differences in the perception of a diagnosis of malignancy in different cultural and socioeconomic environments and, consequently, a potential variability in the resulting level of psychological distress, this information is of importance. In addition, the existence of a link between the two conditions has been recognized in other chronic illnesses; however, information from the HNC patients is scanty, though they have higher levels of psychological disorder and malnutrition than many other patients’ groups [22]. Singer et al. found that at admission and 6 months after admission these patients suffered from symptoms of depression and anxiety 1.5-fold and 2.7-fold, respectively, more frequently than the other patients with cancer [8]. Moreover, in a comparable cohort of patients from our previous research, one third of the patients were found malnourished or even cachectic at diagnosis and this proportion raised to 83.6% at the completion of treatment [19].

To assess the psychological state of our patients, we used the HADS questionnaire with the cut-off sum of 7 points on each of the subscales (HADS-A, HADS-D) to distinguished distressed patients from others [15]. In our group, the prevalence of anxiety was highest before treatment, whereas the HADS-A scores dropped significantly at the time of treatment completion and the same trend was observed 3 months’ post-therapy. The same pattern in score changes was reported by other authors and presumably reflects the anticipatory fear presented before the start of treatment, when a “naive” patient is awaiting treatment that she/he does not know and from which she/he has no idea what to expect. It is, therefore, a fear of the unknown which disappears during the course of treatment [6, 9, 23, 24]. To the contrary and in line with other studies, time variations in the HADS-D scores, although non-significant, showed that depressive symptoms peaked at the end of treatment. The delayed accumulation of symptoms of depression can be attributed to the cumulative effect of fatigue growing during treatment and to other treatment-related side effects to which the patient is exposed and usually increase during the course of treatment, reaching the peak towards its end [6, 9, 23–25].

The nutritional status in our patients was evaluated by a standard set of tests (NRS-2002, anthropometry, laboratory) and using the BIA’s PA measurement, whereas for diagnosing cachectic patients, the international consensus criteria were used [17, 18]. The resulting distribution of the patients in three nutritional categories at each of the assessment points was as expected [11–13, 19], as were the temporal changes in the PA values, pointing to the negative impact of aggressive oncological treatment and related toxicities to nutritional status [26]. In our group, nutritive deterioration occurred despite systematic nutritional counselling and support that was offered to the patients, indicating the limited range of efficiency of these activities. As shown in patients with chronic obstructive pulmonary disease, lower PA values (i.e. below 5.0) indicate not only the presence of muscle weakness and deficit but also the deficits in psychomotor speed and cognitive flexibility, which may also contribute to the lesser efficacy of nutritional counselling [27]. This suggests that a greater efficacy of nutritional therapy could be achieved with appropriate psychological support.

The pioneer research on the interaction of the psychological state and nutritional status in patients with HNC was performed 30 years ago by Westin et al. who demonstrated that depressive patients have a much greater likelihood of developing malnutrition 1 year after treatment completion than those without distress [28]. Until recently there has been little news in this field. In 2012, Britton et al. showed that depression in the first week of treatment with RT was an independent predictor for the development of malnutrition 4 weeks after irradiation, with a higher predictive value compared to some other already established predictive factors, e.g. TNM stage, the use of the feeding tube and the total RT dose [22]. Moreover, the deepening of depression in HNC patients may lead to social isolation to the extent that patients refuse feeding in public [29]. In our cohort, a significant correlation was recorded between nutritional status and HADS-A scores (assessments 1 and 2) as well as HADS-D scores (assessments 2 and 3), with more distressed patients found in the malnourished/cachectic subgroups. The results of PA measurements were less indicative, though at assessment 3, non-anxious patients had significantly higher values of PA than distressed patients. A strong and negative association between anxiety and nutritional status was observed before therapy in lung cancer patients, whereas in HNC patients Van Liew et al. demonstrated that there is a dynamic reciprocal association between depressive symptoms and weight loss over the first year after diagnosis: changes in either variable were associated with concurrent, same-month changes in the other variable [30, 31]. These observations underline the importance of the assessment and treatment of both psychological symptoms and weight loss. However, given that most risk factors for nutritional decline in HNC are non-modifiable (e.g., stage, site, treatment), it is noteworthy that psychological symptoms are treatable, which may lead to an improvement in nutritional status [32].

Our study has several limitations. First of all, the number of included patients was arbitrary determined and small. However, due to the complexity of the study design we correctly predicted that many patients would initially decline their participation or would discontinue co-operation after the initial agreement. Thus, during the 12-month recruitment period, only 49 patients agreed to enter the study, but barely 40 of them completed all three assessments as planned. It is possible that these nine patients had more pronounced symptoms of anxiety/depression and were consequently less motivated to continue their participation in the study. We are aware that small-numbered samples exclude the possibility of analyzing the impact of some important aspects of treatment (e.g. location and extent of surgery) and of using a multivariate analysis to study the independent role of different factors (i.e. age, primary tumor site and stage, treatment characteristics) that may influence the degree of anxiety and depression. In heterogeneous cohorts like ours, such an analysis would be desirable. However, on univariate analysis, the majority of these factors failed to show any impact on either the nutritional or psychological status of the patients; similar observation was reported by Britton et al. [22]. The observed increase of distress recorded by the HADS-D at the end of therapy and a better baseline nutritional status (according to the PA values) in those of our patients treated with primary RT and concurrent chemotherapy were according to expectations as definitive chemoradiation is certainly more burdensome for patients than RT given alone or in an adjuvant setting and should be limited only to well-nourished patients. Thus, the presented results must be interpreted with caution and the study should be considered as a pilot research: a larger study, preferably with the collection of other relevant information (e.g. alcohol consumption, family life and social status, employment, etc.) is warranted.

To conclude, the relationship between the psychological and nutritional status found in this pilot study conducted in a cohort of HNC patients suggests that a larger trial is warranted to clarify the details of the observed associations and identify options for intervention.
